# A population-based study of race-specific risk for placental abruption

**DOI:** 10.1186/1471-2393-8-43

**Published:** 2008-09-12

**Authors:** Tammy T Shen, Emily A DeFranco, David M Stamilio, Jen Jen Chang, Louis J Muglia

**Affiliations:** 1 Department of Pediatrics, Washington University in St. Louis, St. Louis, Missouri, USA; 2 Center for Preterm Birth Research, Washington University in St. Louis, St. Louis, Missouri, USA; 3 Department of Obstetrics and Gynecology, Washington University in St. Louis, St. Louis, Missouri, USA; 4 Department of Community Health in Epidemiology, School of Public Health, Saint Louis University, St. Louis, Missouri, USA

## Abstract

**Background:**

Efforts to elucidate risk factors for placental abruption are imperative due to the severity of complications it produces for both mother and fetus, and its contribution to preterm birth. Ethnicity-based differences in risk of placental abruption and preterm birth have been reported. We tested the hypotheses that race, after adjusting for other factors, is associated with the risk of placental abruption at specific gestational ages, and that there is a greater contribution of placental abruption to the increased risk of preterm birth in Black mothers, compared to White mothers.

**Methods:**

We conducted a population-based cohort study using the Missouri Department of Health's maternally-linked database of all births in Missouri (1989–1997) to assess racial effects on placental abruption and the contribution of placental abruption to preterm birth, at different gestational age categories (n = 664,303).

**Results:**

Among 108,806 births to Black mothers and 555,497 births to White mothers, 1.02% (95% CI 0.96–1.08) of Black births were complicated by placental abruption, compared to 0.71% (95% CI 0.69–0.73) of White births (aOR 1.32, 95% CI 1.22–1.43). The magnitude of risk of placental abruption for Black mothers, compared to White mothers, increased with younger gestational age categories. The risk of placental abruption resulting in term and extreme preterm births (< 28 weeks) was higher for Black mothers (aOR 1.15, 95% CI 1.02–1.29 and aOR 1.98, 95% CI 1.58–2.48, respectively). Compared to White women delivering in the same gestational age category, there were a significantly higher proportion of placental abruption in Black mothers who delivered at term, and a significantly lower proportion of placental abruption in Black mothers who delivered in all preterm categories (p < 0.05).

**Conclusion:**

Black women have an increased risk of placental abruption compared to White women, even when controlling for known coexisting risk factors. This risk increase is greatest at the earliest preterm gestational ages when outcomes are the poorest. The relative contribution of placental abruption to term births was greater in Black women, whereas the relative contribution of placental abruption to preterm birth was greater in White women.

## Background

Placental abruption, defined as premature separation of a normally implanted placenta prior to delivery, results from the culmination of underlying pathophysiologic processes that may either be initiated by a single precipitating event (e.g. premature rupture of membranes), or, more commonly, associated with chronic uteroplacental vascular insufficiency (e.g. chronic hypertension) [1]. Placental abruption complicates 0.8 to 1.0% of pregnancies [2], and the incidence appears to be increasing [3]. Furthermore, histologic evidence of decidual hemorrhage has been noted in 2 to 4% of deliveries, even though most cases are not associated with clinical diagnoses of abruption [4].

Placental abruption, especially marginal or peripheral placental abruption, has also been associated with preterm labor [5]. The incidence of abruption peaks at 24 to 26 weeks of gestation [6]. Furthermore, histologic evidence of old hemorrhage was demonstrated in the placentas of over 50% of women with preterm birth (PTB) in one analysis [7]. Interestingly, there appears to be evidence for heterogeneity in the clinical pathways of placental abruption in term and preterm gestations, with acute inflammation more prevalent at preterm than term gestations, and chronic processes present throughout gestation [8]. Risk factors associated with placental abruption comprise previous abruption (strongest risk factor), mechanical factors (i.e. trauma), chronic hypertension, gestational hypertension, cigarette smoking, cocaine use, preterm premature rupture of fetal membranes (PPROM), multiparity, multiple gestations, advanced maternal age, inherited thrombophilias, and polyhydramnios [3,9-20].

Differences in risk of placental abruption based on ethnicity have also been reported. Placental abruption is more common among African-American women (1 in 595) than among White (1 in 876) or Latin-American (1 in 1423) women [21]. Furthermore, the rate of abruption has increased 92% among Black women between 1979–1981 (0.76%) and 1999–2001 (1.43%), whereas the rate increased by 15% among White women over the same period (0.82% in 1979–1981 to 0.94% in 1999–2001) [3].

The influence of maternal race on the risk for PTB has been demonstrated in many studies [22-24]. Black women who have had a PTB are disproportionately at higher risk for subsequent PTB than White women, and this difference in risk based on ethnicity is not adequately explained by socioeconomic status (SES) or access to health care [22-24]. Since Black maternal race is a risk factor for placental abruption as well as PTB, and placental abruption is associated with PTB, we would expect a greater contribution of placental abruption to the increased risk of PTB in Black mothers. However, epidemiological studies to date that have examined racial disparity in placental abruption at different gestational age categories are lacking.

The Missouri Department of Health's maternally linked birth-death certificate database is a unique and comprehensive resource for assessing birth outcomes across racial, SES and maternal medical risk factors. Using this database to analyze potential racial, SES and medical contributors to the occurrence of placental abruption, we tested the hypothesis that race, while adjusting for other known risk factors, is associated with the risk of placental abruption. Furthermore, we proceeded to estimate the relative contribution of placental abruption to PTB in Black and White mothers, testing the hypothesis that there is a greater contribution of placental abruption to the increased risk of PTB in Black mothers, compared to White mothers.

## Methods

### Study design

The protocol for this study was approved by the Missouri Department of Health and Senior Services, and was exempt from review by the Human Studies Committee of Washington University in Saint Louis and the Missouri Department of Health and Senior Services Institutional Review Board. We developed a study to analyze the Missouri Department of Health's de-identified maternally linked birth-death certificate database, which includes all 1,577,082 live births or fetal deaths in Missouri from 1978 through 1997. This cohort includes 245,136 (15.6%) births to Black mothers and 1,310,462 (83.3%) births to White mothers. Hispanic mothers were incorporated into the major racial categories. Birth certificate data were entered into the database by hospital agents, and were subjected to quality assurance measures. Methods for constructing and evaluating the database with live birth and fetal death records organized into siblingships using probabilistic linkage methods and calculation of weighted scores for every possible pair of records that reflects the likelihood that they belong to the same person have been described [25].

Because our primary interest is to determine racial, SES and maternal medical risk factors associated with placental abruption leading to live births, we excluded fetal deaths in utero. Congenital anomalies and multiple gestation births were also excluded due to their known association with birth complications. Since the cohort analysis compares Black and White racial contributions (i.e. exposures) to placental abruption, births from mothers of other races were excluded. Restriction to Black and White races was based largely on the fact that the prevalence of other races is very low (0.20% Native American, 0.16% Chinese, 0.05% Japanese, 0.01% Hawaiian, 0.15% Filipino, 0.03% other, 0.57% missing) in Missouri, precluding a meaningful analysis of rare outcomes. The analysis was further restricted to live births at gestational ages ≥ 20 and ≤ 44 weeks. Because the rate of missing data prior to 1989 was unacceptably high, we limited the analysis to the years from 1989 to 1997, in which the missing data rate was < 5%. We conducted a population-based cohort study on the remaining singleton live births for the occurrence of placental abruption, and its relation to racial, SES and maternal medical factors.

### Measure

Placental abruption was defined as occurring if coded affirmatively in the database. Maternal race was coded in the database by self-report by patient. PTB as defined by the World Health Organization is delivery at less than 37 weeks gestational age[26]. We focused our analysis for preterm placental abruption to those births occurring at less than 35 weeks in order to avoid borderline gestational ages, which are more prone to misclassification bias, and to identify the population of infants born at the earliest gestations when prognoses are often poor. We defined late PTB as those occurring between 32 and 34^6/7 ^weeks, very PTB as those occurring between 28 and 31^6/7 ^weeks, and extreme PTB as those births occurring at less than 28 weeks of gestation.

The primary exposure was race, including categories Black race, and White race, with White race being the reference group. The primary binary outcome variable was the occurrence of placental abruption. We then created a categorical outcome variable that was the occurrence of placental abruption resulting in birth at various gestational age categories, (1) term or post-term, (2) late PTB, (3) very PTB, and (4) extreme PTB. The reference category was no occurrence of placental abruption across all gestational ages. We also performed stratified analyses examining the risk of placental abruption with race for various high and low risk groups. High risk groups include mothers with low SES, mothers with no prenatal care, mothers who smoked cigarettes during pregnancy, mothers with chronic hypertension, and mothers with gestational hypertension. The low risk group includes mothers with more than 12 years of education, no indicators of low SES (Medicaid, food stamps, or WIC), married status, some level of prenatal care, maternal age between 20 and 35, no gestational hypertension, chronic hypertension, diabetes or renal problems, and no alcohol or cigarette use during pregnancy. Furthermore, we analyzed the occurrence of placental abruption stratified by gestational age. For each gestational age stratum, the binary outcome variable was the occurrence of placental abruption, and the reference category was no occurrence of placental abruption in that gestational age category.

The following factors were used to identify mothers with low SES at the time of delivery: mother was a recipient of state-funded Medicaid assistance, food stamps or the Special Supplemental Nutrition Program for Women, Infants and Children (WIC Program). A binary composite SES variable was created, using the individual dichotomous indicators of low SES, and was defined as low SES if any indicator was present (recipient of Medicaid, food stamps or WIC). A binary variable of low maternal education was created indicating education < 12 years. Maternal age was analyzed as a categorical variable with the following categories: teenage pregnancy (reference), maternal age ≥ 20 years and < 35 years, and advanced maternal age (≥ 35 years). A binary variable was created for lack of prenatal care (derived from a continuous variable that indicated the month of pregnancy at which prenatal care was initiated). We created a continuous variable of maternal pre-pregnancy body mass index (BMI) from maternal height and pre-pregnancy weight, and from this created a categorical variable with low BMI (< 20 kg/m^2^), intermediate BMI (≥ 20 kg/m^2 ^and ≤ 30 kg/m^2^), and high BMI (> 30 kg/m^2^). We created a dichotomous variable for primigravida from the gravidity variable. Other maternal risk factors considered included cigarette smoking, alcohol use, pre-gestational diabetes, chronic hypertension, gestational hypertension, and chronic renal disease.

### Statistical analysis

Data were analyzed using Stata SE 9.2 for Windows (College Station, Texas). For binary outcome variables, unadjusted relative risks were calculated using chi-square tests, and adjusted odds ratios were calculated using binary logistic regression models. For higher-order categorical outcome variables, unadjusted and adjusted odds ratios (aOR) were approximated with the relative risk ratio (RRR) using multinomial logistic regression models. The chi-square test was used to test significance for trend by gestational age (unadjusted). Significant covariates and interaction variables (between race and covariates such as SES) were selected for inclusion in the final multivariable models if there was a 10% or greater difference between the adjusted and unadjusted estimate of the effect, and if the confounding relationship was clinically and biologically important and plausible. All occurrence analyses were adjusted for clustering in siblingships as identified by a unique siblingship number, by which births to the same mothers were identified.

## Results

### Population demographics

The cohort analyzed included 664,303 singleton live births with 108,806 (16.4%) births to Black mothers, and 555,497 (83.6%) births to White mothers. Black mothers, compared to White mothers, delivered at a lower mean gestational age, had a younger mean maternal age, and had a greater proportion of teenage pregnancies. Black mothers were also characterized by a greater proportion having maternal education < 12 years, indicators of low SES, unmarried status, no prenatal care, and obesity (BMI > 30 kg/m^2^). A greater proportion of White mothers, compared to that of Black mothers, was primigravida, had pre-pregnancy BMI < 20 kg/m^2^, and smoked cigarettes during pregnancy, compared to Black mothers. Furthermore, Black mothers were characterized by a greater proportion with chronic hypertension, gestational hypertension, renal disease, and alcohol use during pregnancy. There was no significant difference in proportion of pre-gestational diabetes between Black and White mothers (see Table 1).

**Table 1:** Baseline characteristics by race for individual births (n = 664,303)

**Characteristics**	**Black**	**White**	
		
	**n (%)**	**n (%)**	P*
**Total births**	108,806 (16.4)	555,497 (83.6)	< 0.001
**Mean gestational age at delivery**	38.4 ± 3.1	39.1 ± 2.2	< 0.001
**Maternal age (years)**	23.9 ± 5.9	26.6 ± 5.7	< 0.001
**Maternal age categories**			< 0.001
**Age < 20 years**	28,727 (26.4)	65,785 (11.8)	
**Age ≥ 20 and < 35 years**	73,736 (67.8)	437,468 (78.8)	
**Age ≥ 35 years**	6,326 (5.8)	52,218 (9.4)	
**Maternal education < 12 years**	35,192 (33.0)	94,925 (17.2)	< 0.001
**Indicators of low socioeconomic status†**	86,303 (79.8)	207,159 (37.4)	< 0.001
**Unmarried**	84,362 (77.6)	120,187 (21.7)	< 0.001
**Primigravida**	40,163 (37.0)	231,226 (41.7)	< 0.001
**No prenatal care**	5,094 (4.9)	3,966 (0.7)	< 0.001
**Body mass index categories**			< 0.001
**Body mass index < 20**	17,814 (17.1)	116,300 (21.5)	
**Body mass index ≥ 20 and ≤ 30**	67,678 (65.0)	355,828 (65.8)	
**Body mass index > 30**	18,661 (17.9)	68,875 (12.7)	
**Type I or II Diabetes**	2,480 (2.3)	13,400 (2.4)	0.120
**Chronic hypertension**	1,408 (1.3)	3,932 (0.7)	< 0.001
**Gestational hypertension**	5,570 (5.1)	21,515 (3.9)	< 0.001
**Renal disease**	326 (0.3)	1,258 (0.2)	< 0.001
**Alcohol use**	3,644 (3.4)	11,311 (2.0)	< 0.001
**Cigarette use**	19,340 (17.9)	126,897 (22.9)	0.002

* The p value for a chi-square test, adjusted for clustering for more than one birth to one mother.

† The composite indicator of low socioeconomic status, which includes Medicaid, WIC or food stamps.

Some percentages do not add up due to missing values (less than 5% for any given variable).

The cases of placental abruption included 5,065 births (0.76% of the total, 95%CI 0.74–0.78), including 1,108 (1.02%, 95% CI 0.96–1.08) births to Black mothers, and 3,957 (0.71%, 95% CI 0.69–0.73) births to White mothers. Among cases of placental abruption, Black mothers, compared to White mothers, were more likely to deliver at a younger gestational age, be at a younger age (teenage pregnancy), have < 12 years of education, have indicators of low SES, be unmarried, be multiparous, have no prenatal care, be obese (BMI > 30 kg/m^2^), have chronic hypertension and have used alcohol during pregnancy. A lower proportion of Black mothers reported cigarette use during pregnancy (see Table 2).

**Table 2:** Baseline characteristics by race in cases of placental abruption (n = 5,065)

**Characteristics**	**Black**	**White**	
		
	**n (%)**	**n (%)**	P*
**Number of births with abruption**	1,108	3,957	
**Rate of abruption for births to**	1.0%	0.7%	< 0.001
**Black and White mothers**			
**Mean gestational age at delivery**	34.0 ± 5.4	35.7 ± 4.6	< 0.001
**Maternal age (years)**	25.1 ± 6.3	27.0 ± 6.0	< 0.001
**Maternal age categories**			< 0.001
**Age < 20 years**	235 (21.2)	472 (11.9)	
**Age ≥ 20 and < 35 years**	769 (69.4)	3,028 (76.5)	
**Age ≥ 35 years**	104 (9.4)	457 (11.6)	
**Maternal education < 12 years**	381 (35.4)	804 (20.5)	< 0.001
**Indicators of low socioeconomic status†**	887 (80.8)	1,678 (42.5)	< 0.001
**Unmarried**	879 (79.4)	1,070 (27.1)	< 0.001
**Primigravida**	307 (27.9)	1,444 (36.6)	< 0.001
**No prenatal care**	143 (13.8)	68 (1.8)	< 0.001
**Body mass index categories**			< 0.001
**Body mass index < 20**	217 (21.0)	1,061 (27.9)	
**Body mass index ≥ 20 and ≤ 30**	675 (65.2)	2,322 (61.1)	
**Body mass index > 30**	144 (13.9)	415 (10.9)	
**Type I or II Diabetes**	23 (2.1)	94 (2.4)	0.607
**Chronic hypertension**	29 (2.6)	52 (1.3)	0.001
**Gestational hypertension**	107 (9.7)	316 (8.0)	0.074
**Renal disease**	4 (0.4)	21 (0.5)	0.562
**Alcohol use**	83 (7.6)	118 (3.0)	< 0.001
**Cigarette use**	307 (28.0)	1,364 (34.7)	0.008

* The p value for a chi-square test, adjusted for clustering for more than one birth to one mother.

† The composite indicator of low socioeconomic status, which includes Medicaid, WIC or food stamps.

Some percentages do not add up due to missing values (less than 5% for any given variable).

### Risk of placental abruption associated with race

Black mothers, compared to White mothers, were overall 1.32 times more likely to have placental abruption (95% CI 1.22–1.43). The magnitude of relative risk increase of placental abruption for Black mothers, compared to White mothers, increased as the severity of prematurity worsened (p < 0.001). Black mothers were only slightly more likely to have placental abruption term or post-term (aOR 1.15, 95% CI 1.02–1.29), compared to White mothers, but Black mothers were almost twice as likely to have placental abruption with extreme preterm birth (aOR 1.98, 95% CI 1.58–2.48) (see Table 3).

**Table 3:** Risk of placental abruption in Black compared to White women by gestational age category (categorical analysis)

**Birth Outcome**		**Black**	**White**	**Unadjusted**	Adjusted*
				
	**n (%)**	**n (%)**	**n (%)**	**RR (95% CI)**	**OR (95% CI)**
**Total births**	664,303	108,806	555,497		
**No Abruption**	658,922 (99.19)	107,663 (98.95)	551,259 (99.24)	Reference	Reference
**Abruption†**	5,065 (0.76)	1,108 (1.02)	3,957 (0.71)	1.43 (1.34–1.53)	1.32 (1.22–1.43)
**Abruption and Term Births (≥ 35)‡**	3,310 (0.50)	605 (0.56)	2,705 (0.49)	1.15 (1.05–1.25)	1.15 (1.02–1.29)
**Abruption and Late PTB (32–34)‡**	725 (0.11)	161 (0.15)	564 (0.10)	1.46 (1.23–1.74)	1.29 (1.09–1.53)
**Abruption and Very PTB (28–31)‡**	550 (0.08)	168 (0.15)	382 (0.07)	2.25 (1.88–2.70)	1.92 (1.58–2.33)
**Abruption and Extreme PTB (20–27)‡**	466 (0.07)	170 (0.16)	296 (0.05)	2.94 (2.43–3.55)	1.98 (1.58–2.48)

* Covariates in regression model are unmarried, cigarette use, no prenatal care, chronic hypertension, and gestational hypertension.

† Binary variable, OR was calculated using binary logistic regression.

‡ Categorical variable, OR was approximated with RRR using multinomial logistic regression.

Significant covariates included in the regression model for race and placental abruption were unmarried status (aOR 1.07, 95% CI 1.01–1.14), cigarette use (aOR 1.75, 95% CI 1.65–1.86, no prenatal care (aOR 2.51, 95% CI 2.19–2.87), chronic hypertension (aOR 1.76, 95%CI 1.44–2.15), and gestational hypertension (aOR 2.24, 95% CI 2.06–2.44). Other variables that had a significant effect on placental abruption (but were not part of the final explanatory regression model because they did not alter the estimate of the effect of race) were age 20–30 relative to teenage pregnancy (aOR 1.16, 95% CI 1.06–1.26), advanced maternal age relative to teenage pregnancy (aOR 1.56, 95% CI 1.38–1.76), primigravida (aOR 0.77, 95% CI 0.73–0.81), pre-pregnancy BMI < 20 (aOR 1.33, 95% CI 1.24–1.42), pre-pregnancy BMI > 30 (aOR 0.82, 95% CI 0.75–0.90), renal disease (aOR 1.84, 95% CI 1.30–2.60), and alcohol use (aOR 1.30, 95% CI 1.13–1.49).

When we examined the risk of placental abruption associated with race in subgroups of women selected for various high or low risk characteristics, overall we found that these subsets of Black women had an increased risk of placental abruption, compared to the same subsets of White women. In the subgroup of women positive for indicators of low SES (n = 293,386), Black women had a 30% increase in risk of placental abruption, compared to White women (RR 1.27, 95% CI 1.17–1.38). Black women who had no prenatal care also had a 60% increase in risk of placental abruption, compared to White women who also had no prenatal care (n = 9,042, RR 1.63, 95% CI 1.23–2.17). Black smokers also had an increased risk of placental abruption compared to White smokers (n = 146,198, RR 1.48, 95% CI 1.31–1.67). In the subgroup of women with chronic hypertension, Black women did not have a statistically significant increase in risk of placental abruption, compared to White women (n = 5,340, RR 1.56, 95% CI 0.99–2.44). Black women who had gestational hypertension had a higher risk of PPROM, compared to White women with gestational hypertension (n = 27,075, RR 1.31, 95% CI 1.05–1.63). In the low-risk subgroup of women with no major SES or medical risk factors (n = 223,780), low-risk Black women also had an increased risk of placental abruption, compared to low-risk White mothers (RR 1.45, 95% CI 1.13–1.87).

### Relative contribution of placental abruption to preterm birth

For the subset of women delivering at term or post-term gestational ages, there was a significantly greater proportion of placental abruption in Black mothers (0.61%), compared to White mothers (0.50%) (p < 0.001). In contrast, the proportion of Black mothers with placental abruption delivering at late preterm, very preterm, or extreme preterm birth gestation ages was lower than White mothers (see Table 4). The frequency of placental abruption in these preterm birth categories increased as gestational age at birth decreased for both Black mothers and White mothers (see Table 4).

**Table 4:** Relative contribution of placental abruption to preterm birth in Black compared to White women (stratified analysis)

**Birth Outcome**		**Black**	**White**	P*
		
	**n (%)**	**n (%)**	**n (%)**	
**Abruption and Term Births (≥ 35)†**	3,310	605	2,705	< 0.001
**Number of Term Births**	635,772	99,213	536,559	
**Frequency of Abruption**	(0.52)	(0.61)	(0.50)	
				
**Abruption and Late PTB (32–34)†**	725	161	564	< 0.001
**Number of Late PTB**	16,292	5,128	11,164	
**Frequency of Abruption**	(4.45)	(3.14)	(5.05)	
				
**Abruption and Very PTB (28–31)†**	550	168	382	0.015
**Number of Very PTB**	6,869	2,483	4,386	
**Frequency of Abruption**	(8.01)	(6.77)	(8.71)	
				
**Abruption and Extreme PTB (20–27)†**	466	170	296	0.029
**Number of Extreme PTB**	3,929	1,658	2,271	
**Frequency of Abruption**	(11.86)	(10.25)	(13.03)	

* The p value for chi-square test.

† Binary variable restricted to subpopulations of term births, late PTB, very PTB, and extreme PTB, OR was calculated using binary logistic regression.

## Discussion

In this study, we examined the association among placental abruption, preterm birth and maternal race. We found that self-reported Black maternal race, compared to White race, was significantly associated with an increased risk of placental abruption, even after adjusting for SES and maternal medical risk factors. These findings confirmed previous epidemiological studies showing increased risk of placental abruption in Black mothers, compared to White mothers [3,9,21]. We also observed that the relative risk increase for placental abruption for Black mothers was greater at earlier gestational age categories, compared to White mothers.

Since we confirmed Black race to be a risk factor for placental abruption, and both Black race and placental abruption have been identified as risk factors for PTB, we expected a greater contribution of placental abruption to the increased risk of PTB in Black mothers. However, we did not find that to be the case. We found that for the subset of women delivering preterm, there was a significantly lower proportion of placental abruption in Black mothers, compared to White mothers. Conversely, for the subset of women delivering at term, there was a significantly higher proportion of placental abruption in Black mothers, compared to White mothers.

While this result may seem initially contradictory to the finding that Black women, compared to White mothers, were at an increased risk of placental abruption, and that there was a trend with severity of prematurity, this perspective highlights preterm birth frequency issues. Firstly, numerous studies have shown that Black mothers, compared to White mothers, are at significantly higher risk of PTB (and recurrence), even after adjustment for important SES and maternal medical risk factors [22-24]. This study shows that specific mechanisms of PTB other than placental abruption, such as spontaneous preterm labor (SPTL) and PPROM, may have much greater contributions to the increased risk of PTB in Black women, compared to the mechanism of placental abruption.

Secondly, the fact that Black women have a greater proportion of placental abruption in term pregnancies, but have a lesser proportion placental abruption in preterm pregnancies, may hint at different causative pathways at preterm and at term gestations that culminate in placental abruption. Evidence from previous studies suggests that placental abruption is the manifestation of at least two distinct clinical pathways: 1) acute inflammation-associated pathways (such as premature rupture of membranes, etc.), and 2) chronic clinical processes (such as chronic hypertension, gestational hypertension, diabetes, smoking, etc.) [7,8,16,27]. At preterm gestations, placental abruption seems to be more frequently associated with acute inflammation, notably PPROM, whereas chronic clinical processes seem to be associated with an increased risk, both at term and preterm births[8].

Finally, the association between placental abruption and maternal race, especially abruption-associated PTB, prominent even after controlling for SES and maternal medical risk factors, may suggest the possibility of a genetic contribution along with environmental components to the pathogenesis of placental abruption. Self-reported race in general accurately reflects ancestry, but the heterogeneity of nativity in Black mothers have also been shown to influence birth outcomes [28,29]. Thus, self-reported race is a reasonable, but not perfect, correlate for ancestry and genetics. However, we also acknowledge that unmeasured confounding environmental risk factors must be considered, and may contribute much of the disparity we observed.

Placental abruption is a distinct and dependent mechanism of PTB. Placental abruption, PPROM, and SPTL have overlapping causes, and probably similar biochemical pathways. Although evidence for environmental contribution for PTB is compelling, there has been increasing evidence for genetic contributions to PTB. Family-based, twin, and ethnic-comparison studies have all suggested that genetics may play a role in PTB, in addition to environmental factors [22,24,30-33]. Several recent candidate gene studies for PTB have also supported the case for genetic contributions [34-38]. Unfortunately, most of the genetic studies in placental abruption have concentrated on polymorphisms in various coagulation genes only, and little or no information is available in African populations or those of African descent [39].

The limitations of our study are typical to those of large, population-based studies. Possible sources of bias concerning measurement error in the database comprise recall, underreporting, miscoding, misclassification and information bias. Recall bias, such as the underreporting of social habit variables (i.e. cigarette smoking, alcohol use, and illicit drug use) and inaccurate reporting and underreporting of prenatal care, weight, past medical history and obstetrics complication variables, likely results in bias towards the null since bias is most likely nondifferential across race. Preterm birth was defined at less than 35 weeks of gestation, in order to decrease the effects of miscoding error and misclassification bias of borderline gestational ages. Information bias may include lack of data on certain maternal medical co-morbidities. Cocaine use is an important contributor to placental abruption, but is not a coded variable in the database. However, it is not likely to influence race-specific effects on placental abruption, as the reported frequency of cocaine use in complicated deliveries is extremely low [40]. We chose to exclude fetal deaths in utero, because it likely represents a group of complicated births having different pathological mechanisms unrelated to placental abruption. Even though the most severe cases of placental abruption (causing death) may be excluded, the effect of that on the relationship between maternal race and placental abruption should be limited. Furthermore, placental abruption may be underestimated in term and post-term gestations, shifting cases of placental abruption toward a lower median gestational age at delivery, but we do not anticipate this shift to be biased by maternal race. Finally, because the racial distribution of Missouri consisted of mainly Black and White races, it precluded analysis of adverse birth outcomes in other races. The population-based nature of the study offers generalizability to a broad spectrum of clinical populations. The large sample size also permits sufficient statistical power for subgroup analysis of a relatively rare outcome in various gestational age categories. More importantly, the results have broad research and clinical implications.

## Conclusion

We find that Black women are at increased risk for placental abruption, especially at early gestational ages, but placental abruption makes up a smaller proportion of causes of PTB for Black women. In addition, the difference in relative contribution of placental abruption between term and preterm gestations suggests heterogeneity in clinical pathways between these two importantly different birth outcomes.

## Competing interests

The authors declare that they have no competing interests.

## Authors' contributions

TTS has made substantial contributions to conception and design, analysis and interpretation of data, and have been involved in drafting the manuscript and revising it critically for important intellectual content. EAD has made substantial contributions to conception and design, and has been involved in revising the manuscript critically for important intellectual content. JJC and DMS participated in analysis and interpretation of data, as well as in revising the manuscript critically for important intellectual content. LJM has made substantial contributions to conception and design, and has been involved in revising the manuscript critically for important intellectual content. All authors read and approved the final transcript.

## Pre-publication history

The pre-publication history for this paper can be accessed here:


